# The Evolution of Joint Surgery

**Published:** 1910-12-31

**Authors:** 


### SPECIAL ARTICLES.

### THE EVOLUTION OF JOINT SURGERY.

THE treatment of tuberculous bones and joints has passed through three stages in its evolution. In the first, during the period when the causes of inflammation were unknown, rest was the only method of treating these lesions. As John Hilton said, "Rest, local rest, by fixing the limb in a proper splint, aided by time, Nature's powerful co-adjutor." During this period a masterly inactivity as regards operation gave the best results. With the advent of antisepsis the pendulum swung out to the extreme, and early interference was advocated in all cases. We are now in the third stage: it consists mainly in the return to the medium by applying asepsis to the older methods of rest and appliances, aided by the further knowledge gained from modern pathology. In the early period, when once an abscess formed it became infected either by bursting or by being opened. Long-continued suppuration then resulted, with the formation of intractable sinuses. The patient was fortunate to recover with a joint fixed in a position in which the limb could be used. Marked deformity was not uncommon, while loss of limb and of life occurred in a large proportion of cases. During this period excision was sometimes attempted, though the results were often disastrous. John Hilton, the great advocate of conservative measures, advised deferring amputation till late; he distinguished between joint abscesses that should be opened and those which might dry up if left to themselves.

The passage to the second period was primarily a stage in conservatism. When a reasonable chance of obtaining a primary union had been afforded to the surgeon by his asepsis, it was argued that by removing at one operation all the diseased tissue the later and more serious results of tuberculous joints would be done away with, and in this way many limbs and lives would be saved. Experience, however, showed that this removal of all the diseased structures was not always possible. Either anatomical reasons prevented a sufficiently wide removal of the disease, or the limits of the disease could not be recognised by the naked eye. In other cases, again, where the whole disease was removed the deformity resulting was found to be greater than when the limb recovered without excision. A further stage of conservatism was then reached, and the operation of arthrectomy or arthrodesis was evolved. This consisted in removing at an early stage all the involved synorial membrane, in scraping or gouging away any superficially involved bone without cutting through or removing any normal bone or cartilage. From this to the third stage was but a step, and that step was the return to a masterly inactivity, with operation only when such was absolutely and imperatively necessary.

* *Diseases of the Joints and Spine.* By Howard Marsh and C. Gordon Watson. Third edition, pp. 632. London: Cassell and Co. 10s. 6d. net.

Entirely different is the aspect now, when we are forced to turn to operation, from what it was forty years ago! In those days it meant the certain infection of the joint. It meant an undoubted period of severe illness to the individual, and always the possibility of death in a short time. Now a tuberculous abscess can be opened and drained without fear of infection; it can be sewn up again so that the patient is not put to the risks of sepsis, dependent on the drainage of the joint.

It may be well, then, to epitomise the principles upon which a tuberculous joint is now treated. The old principle of rest is still the most important. In this we may add that the earlier the rest is started the more efficient it becomes; and in most cases, taken early, it is all that is necessary. Hence we see the value of early diagnosis. This is the first place in which the bacteriologist can now help us; and, indeed, it is perhaps the one place in which he is most valuable. There are to-day three tests—the cutaneous, the conjunctival, and the sub-cutaneous, each of which has but a small percentage of error in diagnosis. Having begun the period of rest, the next principle to bear in mind is that the treatment is intended to get rid of the disease, and that any other consideration is secondary to this. Questions of a mobile joint or a stiff one are at this period not to be thought of. When the disease is cured, a mobile, or partly mobile, joint may be present, and is an undoubted advantage; but to aim at a moveable joint is but to imperil the success of the treatment. The third principle is to operate as seldom as possible, and when operation is necessary, to do as little as possible. If the first two principles that we have laid down be followed, the number of cases that go on to abscess formation will be found to be appreciably diminished. But, unfortunately, many cases do not come under the doctor's care until an abscess is already present. Even then it is not always necessary to operate; certain of these abscesses will eventually become absorbed when treated by rest. When, however, they are nearing the surface they should be opened in order to prevent the risks attendant upon nature's surgery. On this point, opening the different planes of structures covering the abscess at different levels is of great value. This was advocated by Mr. F. J. Steward (*B.M.J.*, January 1907), and it has the advantage of the superficial layers healing before there is any chance of the re-formed tuberculous pus spreading through to the surface. Strict asepsis must be the rule in all such operations. Excision should be left for those cases where suppuration has supervened, and then only so much bone should be removed as will secure efficient drainage.

These, then, are the principles for treating tuberculous joints. There are, however, adjuncts which must not be neglected. Firstly, there is attention to general health, by repairing decayed teeth, by correcting constipation, and by such fresh air and feeding as the means of the patient admit. Secondly,

Bier's treatment may be used, the application of a bandage for six hours out of the twelve is a valuable help in these cases.

Lastly, we may mention injections. Of these two forms are used. The injection of a sterilised emulsion of iodoform in glycerine into the joint is much advocated by Mr. Waterhouse (B.M.J., July 1908), who advises it to be done once a month. The other form is injection of tuberculin; this must be carefully regulated by the opsonic index, and is an important adjunct to treatment.

Just as John Hilton's "Rest and Pain" was the book which typefied the best form of the old treatment, so Mr. Howard Marsh's "Diseases of the Joints and Spine" is the best exponent of the new. After fifteen years Professor Marsh has, with the help of Mr. Gordon Watson, brought out a new edition. Professor Marsh was one of the earliest to see the advantage of this treatment. He has consistently supported it, until he has proved it to be the best form. The book is therefore a valuable one, both in giving many very helpful details, and in showing the evolution of this branch of surgery which we have briefly traced above.

